# Thermo-Responsive Hydrogels Encapsulating Targeted Core–Shell Nanoparticles as Injectable Drug Delivery Systems

**DOI:** 10.3390/pharmaceutics15092358

**Published:** 2023-09-21

**Authors:** Elif Gulin Ertugral-Samgar, Ali Murad Ozmen, Ozgul Gok

**Affiliations:** 1 Medical Engineering Program, Graduate School of Natural and Applied Sciences, Acibadem Mehmet Ali Aydinlar University, 34752 Istanbul, Turkey; elif.ertugral@live.acibadem.edu.tr (E.G.E.-S.); alimurad93@gmail.com (A.M.O.); 2 Department of Biomedical Engineering, Faculty of Engineering and Natural Sciences, Acibadem Mehmet Ali Aydinlar University, 34752 Istanbul, Turkey

**Keywords:** core–shell nanoparticles, drug-loaded scaffolds, injectable hydrogels, thermo-responsive polymers, controlled drug release

## Abstract

As therapeutic agents that allow for minimally invasive administration, injectable biomaterials stand out as effective tools with tunable properties. Furthermore, hydrogels with responsive features present potential platforms for delivering therapeutics to desired sites in the body. Herein, temperature-responsive hydrogel scaffolds with embedded targeted nanoparticles were utilized to achieve controlled drug delivery via local drug administration. Poly(N-isopropylacrylamide) (pNIPAM) hydrogels, prepared with an ethylene-glycol-based cross-linker, demonstrated thermo-sensitive gelation ability upon injection into environments at body temperature. This hydrogel network was engineered to provide a slow and controlled drug release profile by being incorporated with curcumin-loaded nanoparticles bearing high encapsulation efficiency. A core (alginate)–shell (chitosan) nanoparticle design was preferred to ensure the stability of the drug molecules encapsulated in the core and to provide slower drug release. Nanoparticle-embedded hydrogels were shown to release curcumin at least four times slower compared to the free nanoparticle itself and to possess high water uptake capacity and more mechanically stable viscoelastic behavior. Moreover, this therapy has the potential to specifically address tumor tissues over-expressing folate receptors like ovaries, as the nanoparticles target the receptors by folic acid conjugation to the periphery. Together with its temperature-driven injectability, it can be concluded that this hydrogel scaffold with drug-loaded and embedded folate-targeting nanoparticles would provide effective therapy for tumor tissues accessible via minimally invasive routes and be beneficial for post-operative drug administration after tumor resection.

## 1. Introduction

Hydrogels are cross-linked, porous networks of hydrophilic polymer chains that serve as outstanding and effective 3D platforms with high water uptake capacity [1]. They allow the conjugation of various therapeutics, like drug molecules and peptide-based biomolecules, to either end groups or side chains due to the presence of functional moieties, such as amine, hydroxyl, carboxylic acid, maleimide, etc., in their structures [2,3,4,5]. Although their physicochemical properties are mainly affected by their swelling ratio for controlled delivery of encapsulated therapeutic agents inside, chemically or physically cross-linked smart polymers also contribute to the development of hydrogels with diverse stimuli responsiveness to temperature, pH, biomolecules, light, magnetic fields, etc. [6,7,8,9]. The drug release profile of these “intelligent hydrogels” is modulated by their polymer chain length, biodegradation rate, cross-linking degree, and pore size as well as where the drug molecules are encapsulated and stabilized against the host environment with regard to the presence of specific enzymes or pH value [10,11]. However, obtaining precise control over the pore size and biodegradation of hydrogel networks might emerge as a serious issue in obtaining controlled release profiles in the presence of a recurring and metastatic cancer type like ovarian cancer or glioma [12,13,14,15,16]. In particular, natural and hydrophilic pH-responsive polymers, such as alginate, chitosan, and gelatin, were shown to liberate internally loaded active agents more rapidly due to their high degradation rate in tumor environments [17,18,19] compared to polyanhydride- and polyester-based synthetic polymers (i.e., poly(glycolide-co-sebacate) (PGS), poly (caprolactone) (PCL), and poly (lactide-co-glycolide) (PLGA)) [20,21,22,23,24]. Thus, an urgent need has evolved for hydrogels with tunable properties for the slower and sustained release of therapeutic agents. Notably, recent literature revealed that the bioavailability and pharmacokinetic features of drug molecules can be improved by their incorporation into hydrogel systems via indirect methods, which mainly benefit from developments in nanotechnology [25].

Nanoparticle-embedded hydrogel scaffolds, which are also known as NP/gel systems, present innovative combinations that have emerged as powerful tools to provide more advantageous hybrid platforms for novel drug delivery systems [26]. The most recent literature covers many studies regarding the incorporation of nanoparticles into hydrogel systems to obtain more tunable drug release behavior [27,28,29]. Combinations of various types of nanoparticles, such as polymeric and metallic ones, as well as nanogels, liposomes, carbon nanotubes, and dendrimers into 3D hydrogel scaffolds have demonstrated the ability to provide not only enhanced drug loading efficiency but also different routes of administration via injectable hydrogels [30,31,32,33,34,35]. Natural polymers like polysaccharides (alginate, hyaluronic acid, chitosan, etc.) are mainly preferred for their excellent biocompatibility and hydrophilicity, whereas the incorporation of synthetic polymers, including PLGA and poly (lactide) (PLA), is more popular due to their controlled biodegradation ability and slow drug release tendency [36,37,38,39]. Their incorporation into polymeric scaffolds like hydrogels has been shown to improve not only the pharmacokinetic profiles of loaded drug molecules but also to enhance their stability and increase local drug concentration, allowing for a drug reservoir at the site of administration [40,41].

The diverse features provided by these multi-component NP/gel systems not only allow for increased local drug concentration at the site of injury after hydrogel implementation but also for the selective accumulation of drug molecules in nanoparticles due to the enhanced permeation and retention (EPR) effect. This effect is dependent upon nanosize or active targeting via the conjugation of a targeting unit like peptides (RGD (Arginylglycylaspartic acid), TAT (YGRKKRRQRRR), monoclonal antibodies, or ligands (folic acid, mannose) to the periphery of these nanoparticles [42,43,44,45,46]. These studies point out the superior effect of NP/gel systems in modulating drug release profiles over an extended period [28,47]. In particular, temperature-responsive hydrogels based on poly(N-isopropylacrylamide) (pNIPAM), poly(N,N-diethylacrylamide) (PDEAM), poly(2-(dimethylamino)ethyl methacrylate) (PDMAEMA), PEG methacrylate polymers (PEGMA), etc. are preferred as the main constructs for the preparation of injectable gels to slow the clearance rate of nanoparticles in the body, resulting in an improved bioavailability for therapeutic agents, such as chemotherapy drugs, antioxidants, anti-inflammatory drugs, and vaccines [48,49,50,51]. To the best of our knowledge, there is no example of a NP/gel system containing a targeted nanoparticle for delivering drug molecules to specific cells.

Therefore, we designed a novel drug-loaded nanoparticle system with a thermo-sensitive hydrogel scaffold to obtain an injectable biomaterial platform for a more controlled and slow drug release profile that could be administered in a minimally invasive manner to the injury site. For this purpose, pNIPAM-based thermo-responsive polymers were utilized to obtain an injectable hydrogel system, which was stabilized by an ethylene-glycol-based cross-linker. Employing polymeric core–shell type nanoparticles as carriers for the anti-inflammatory drug curcumin, this NP/gel system successfully increased the drug loading capacity and demonstrated improved viscoelastic properties. Visually observed in the pores of the hydrogel scaffold, nanoparticles were successfully embedded inside the NP/gel system. Remarkably, this nanoparticle encapsulated hydrogel system resulted in a drug release profile that was four times slower compared to that of the free nanoparticle system. These nanoparticles comprised a core (alginate) and shell (chitosan) made from natural and pH-responsive polymers. The drug molecules were encapsulated at the core and were engineered to deliver the payload to the folate receptor over-expressing cells (such as ovarian and breast cancer) by being conjugated to folic acid molecules on the surface. Thus, we prepared an injectable nanoparticle-embedded hydrogel scaffold with a thermo-responsive feature that demonstrated rapid gelation, which can contribute to wound closure of damaged tissue, and embedded targeted polymeric nanoparticles to obtain the slow, controlled, and pH-responsive release of loaded drug molecules.

## 2. Materials and Methods

### 2.1. Materials

Alginic acid sodium salt from brown algae (low viscosity, Sigma-Aldrich A1112 (St. Louis, MO, USA), average molecular weight of 30,000–100,000), calcium chloride, sodium dodecyl sulfate (SDS), chitosan (low molecular weight [LMW], 50–190 kDa, 75–85% deacetylated); (high molecular weight [HMW], 310–375 kDa, >75% deacetylated), curcumin (from Curcuma longa (Turmeric) powder), and Tween 80 (for synthesis) were purchased from Sigma-Aldrich for the preparation of the nanoparticles. Poly(N-isopropyl acrylamide) (pNIPAM, Mwt: 40kDa), N,N,N′,N′-Tetramethylethylenediamine (TEMED, bioreagent, suitable for electrophoresis 99%), triethylene glycol dimethacrylate (containing 80–120 ppm inhibitor, 95%), folic acid (FA, 97%), diisopropylamine (DIPA, 99.5%), N,N Dimethylformamide (DMF, anhydrous, 99.8%), ammonium persulfate (APS, for molecular biology and for electrophoresis 99.8%), and poly-L-lysine (0.1% (*w*/*v*) in H_2_O)) were purchased from Sigma-Aldrich for the preparation of the hydrogel scaffold. Aluminum oxide (90 active basic [0.063–0.200 mm] [activity stage I] for column chromatography) was purchased from Merck and used to filter the inhibitor in triethylene glycol dimethacrylate, as received. Acetate buffer (pH 5.5) was prepared with acetone and acetic acid (Merck, Rahway, NJ, USA) into distilled water, and phosphate saline buffer tablets (Biomatik Corporation, Kitchener, ON, Canada) were dissolved in distilled water; both solutions were used for the drug release profile experiments. Hexafluorophosphate azabenzotriazole tetramethyl uronium (HATU, 97%, Rahway, NJ, USA), diethyl ether, and sodium sulfate (anhydrous for analysis) were purchased from Merck. Spectrum™ Spectra/Por™ 6 (Pre-wetted Standard RC Dialysis Tubing, 1 kD MWCO) was purchased from Fisher Science (Waltham, MA USA) for dialysis.

### 2.2. Preparation of Alginate Nanoparticles (AA-NPs)

The empty (without drug) alginate nanoparticles were prepared according to procedures described in the literature with slight modifications [52]. Briefly, low viscosity sodium alginate (0.5% by weight) was physically cross-linked with calcium chloride at a ratio of 19:1 by volume for 2 h at room temperature to obtain nanoparticles. After 2 h, the mixture was centrifuged at 14,000 rpm for 30 min and rinsed with ultrapure water, followed by another centrifugation at 5000 rpm for 5 min. This washing step was repeated twice. The supernatant was decanted and filtered with a 0.22 µm cellulose acetate syringe filter for further measurements regarding the characterization of prepared nanoparticles. Both 50 and 75 mM CaCl_2_ solutions were utilized for the cross-linking of alginate polymers; higher concentrations of Ca^+2^ ions seemed to fasten cross-linking to yield aggregates. Additionally, nanoparticles were stabilized by ionic balance obtained with the help of a commonly used surfactant, SDS, and a polycationic agent, poly-l-lysine, in differing amounts (Table 1).

### 2.3. Preparation of Curcumin-Loaded Alginate Nanoparticles (AA-Cur-NPs)

Curcumin-loaded alginate nanoparticles were prepared according to the procedure used to create the empty (without drug) nanoparticles but with the addition of 1 mg/mL curcumin (prepared in absolute ethanol) to the polymer solution [53]. After obtaining nanoparticles, the previously described centrifugation and washing steps were repeated, and the supernatant was collected and filtered with a 0.22 µm cellulose acetate syringe filter for further characterization measurements.

### 2.4. Preparation of Chitosan-Coated Curcumin-Loaded Alginate Nanoparticles (CS[AA-Cur-NPs])

The curcumin-loaded alginate nanoparticles were coated with the chitosan polymer according to the procedures described in previous literature with slight modifications [54]. Briefly, curcumin-loaded alginate nanoparticles were added dropwise into a 0.1% *w/v* (weight/volume) chitosan (LMW) solution by micropipette to achieve a chitosan to nanoparticle (CS:NP) ratio of 1:2 by *v*/*v* (volume/volume). The mixture was stirred magnetically for 2 h at room temperature. After 2h, the mixture was centrifuged at 14,000 rpm for 30 min and rinsed with ultrapure water (ddH_2_O); then, it was centrifuged at 5000 rpm for 5 min. This step was repeated twice. Finally, the supernatant was collected and filtered with a 0.22 µm cellulose acetate syringe filter for further characterization measurements. The same procedure was utilized to coat the drug-loaded, alginate-based nanoparticles.

### 2.5. Conjugation of Folic Acid (FA) on the Surface of Nanoparticles (FA-CS[AA-Cur-NPs])

FA-conjugated NP synthesis was performed according to the procedure described in the literature [55]. First, HATU (3.9 mg, 10 µmol), FA (4.1 mg, 9.29 µmol), and DIPA (0.68 µL, 9.31 µmol) were dissolved in 1.5 mL of anhydrous DMF solution and stirred magnetically for 2 h at room temperature to activate the beta-carboxylic acid in FA. This solution was then transferred onto the nanoparticle powder, which was obtained by lyophilization of 1 mL of nanoparticle solution in a round-bottom flask. Suspended in DMF solution, the amine groups in the chitosan on the nanoparticle surface underwent the FA-conjugation reaction overnight and at room temperature. The next day, dialysis was performed against ddH_2_O to remove free FA molecules and excess DMF in the nanoparticle solution.

### 2.6. Characterization Methods for Prepared Nanoparticles

Regarding the hydrodynamic volume measurement, the size determination of the nanoparticles was obtained using dynamic light scattering (DLS) measurements in water at 25 °C using the AntonPaar Litesizer500 and Wyatt Technologies Dynapro Nanostar. Hydrodynamic diameter and polydispersity index of nanoparticles (AA NP, Cur-AA NP, and CS(Cur-AA NP)) were measured in ddH_2_O and at room temperature. A total of 1 mL of these solutions was transferred into a glass cuvette and run for 10 processes with 5 s equilibrium time for three repetitions.

The surface charge measurement was performed via the Anton Paar Litesizer500 at 25 °C with 100 runs for three repetitions. The morphology of obtained nanoparticles was evaluated with the aid of a transmission electron microscopic (TEM) (Thermo Fisher Scientific, Waltham, MA USA, Talos L120C) operated at 20 kV with 3.5 spot size; support–copper TEM grids were utilized to visualize nanoparticles. Freeze-dried nanoparticles were investigated for their primary functional groups via Fourier transform infrared (FT-IR) spectroscopy (Thermo Fisher Scientific Inc.; Nicolet 380, Madison, WI, USA). The stability of the nanoparticles was assessed according to the day-by-day change in hydrodynamic diameter obtained by the DLS instrument as described above. The hydrodynamic diameter of nanoparticles was run for 10 successive runs with a 5 s equilibrium time for three repetitions. Size measurements were taken at different time intervals for 10 days [56].

### 2.7. Determination of Drug Encapsulation Efficiency

Encapsulation efficiency values for drug-loaded nanoparticles were determined using liquid chromatography–mass spectrometry/mass spectrometry (LC-MS/MS) (Agilent Technologies (Santa Clara, CA, USA) 1260 Infinity II) [57]. Briefly, the multiple-reaction monitoring (MRM) method specific to the curcumin molecule was created in the LC-MS/MS instrument by taking its molecular ion peak with an *m/z* value of 365.2 gmol^−1^. The standard curve of curcumin was plotted using this MRM method with serially diluted samples at concentrations of 25, 50, 100, 200, 500, 1000, 2500, 5000, 7500, and 10,000 ppb. The C18 column was used as the stationary phase, and 50%ACN:50%ddH_2_O was passed through with a flow rate of 0.5 mL/min. To measure the amount of drug molecules loaded in the nanoparticles, dialysis-purified and drug-loaded nanoparticles were incubated in a DMF/water mixture at a ratio of 1:1:4 (NP:DMF:dH_2_O) to dissociate and liberate the drug molecules in the solution. The amount of released curcumin was determined by the prepared calibration curve. For the drug-loaded nanoparticles, drug encapsulation efficiency (DEE) values were determined based on the following Equation (1):

DEE: [mass of drug_(loaded)_/mass of drug_(feed)_] × 100 (1)

### 2.8. Preparation of Nanoparticle-Embedded Hydrogel Scaffolds (HG/CS(AA-NPs))

Nanoparticle (with or without drug)-embedded hydrogels were prepared by the radical-based gelation of a dimethacrylate-containing monomer, into which the thermo-responsive polymer chains pNIPAM were entrapped and solidified due to the temperature increase (37 °C) above its T_g_ value (32 °C) to provide an interpenetrating hydrogel scaffold. In detail, the nanoparticle solution was added into a 0.14% pNIPAM (*w*/*v*) (Mwt: 40 kg mol^−1^) solution at a 1:4 ratio (*v*/*v*). Al_2_O_3_-filtered tetraethylene glycol dimethacrylate (20.5 µL, 57.3 µmol) was mixed with the pNIPAM solution, and 20 µL of 5% (*w*/*v*) APS and 30 µL of TEMED (0.20 µmol) were added into the polymer mixture to start the cross-linking process. After vigorous vortexing, gelation was clearly observed. However, to characterize a completely gelated hydrogel structure, this mixture was incubated in a thermal shaker at 37 °C overnight. The blank hydrogel was prepared as a control using the same amount of dH_2_O instead of the nanoparticle solution [58].

### 2.9. Characterization of Nanoparticle-Embedded Hydrogel Scaffolds (HG/CS(AA-NPs))

The degradation, swelling behavior, thermo-responsive (injectability) property, and morphology of nanoparticle-embedded hydrogel scaffolds were investigated using various techniques [59]. For the morphological analysis, scanning electron micrography (SEM) images were obtained using Thermo Fisher Scientific Quanta 650 FEG. SEM samples were coated with 20 nm of gold (Au/Pb) under vacuum and fixed on the stub with carbon tape. Regarding the mechanical analysis, the rheological properties of the hydrogels were assessed using the Malvern Kinexus rheometer. The gelation process and degradation properties of the hydrogel were evaluated using J2 SR 4703 SS geometry. The degradation property was studied by taking measurements at parameters between γ = 0.001 and γ = 1 at f = 1 Hz and 37 °C (30 data points). The gelation process was analyzed under CD-auto strain mode with γ = 0.01 at f = 1 Hz at 37 °C (30 data points.) The strain-dependent oscillatory rheology and frequency-dependent oscillatory rheology of hydrogel scaffolds were tested at 0.1 Hz, 50.5 Hz, and 100 Hz, with γ = 0.01, at 37 °C. The thermo-responsive property of the hydrogels was investigated with the use of a small piece of freshly obtained chicken breast tissue (4 cm × 4 cm). Briefly, a small scratch (1 cm) was made on the tissue surface to mimic a wound. The tissue sample was placed in a thermal shaker adjusted to 37 °C. Then, the mixture prepared for hydrogel formation was immediately injected into the scratch site with a syringe and allowed to spread. The tissue sample was then stored in the thermal shaker at 37 °C for further observation of the gelation process and wound closure [60,61]. The swelling behavior of the hydrogels (with and without nanoparticles) was investigated by calculating the water uptake percentage as a function of time until the equilibrium condition was observed. Briefly, a swollen piece of hydrogel was frozen at −20 °C, freeze-dried in a lyophilizer, then incubated in dH_2_O. At different time points, the weight of the swollen hydrogel was measured. An increase in the weight of hydrogel was recorded as a function of time. Obtained data were used to plot the graphs showing swelling capacity. The swelling ratio, W, was calculated via Equation (2), where m_w_ and m_d_ are the weights of wet and dry samples, respectively.

W (%) = ((m_w_ − m_d_)/m_d_) × 100 (2)

### 2.10. Drug Release Studies

Both targeted nanoparticles and nanoparticle-embedded hydrogels were analyzed for their drug release profiles at different pH values via the dialysis method with respect to time. The dialysis set-up utilized 10 mM of phosphate buffered saline (PBS, pH 7.4) and sodium acetate buffer (SAB, pH 5.5) as receptor solutions in the drug release experiments to mimic the human body conditions of healthy tissue and tumor microenvironment, respectively [62]. Dialysis membranes (Mwt cut-off: 1 kDa) containing either nanoparticles or NP-embedded hydrogels were placed in a thermal shaker and the temperature was adjusted to 37 °C. Dialysis set-ups were subjected to continuous shaking at 200 rpm. At different time intervals (0, 0.5, 1, 2, 4, 6, 8, 10, 24, 48 h, and so on), 50 µL from the receptor solutions was withdrawn and analyzed for its free drug content using the LC-MS/MS according to the MRM method, prepared specific to curcumin.

## 3. Results and Discussion

### 3.1. Preparation of Drug-Loaded Nanoparticles

Alginate nanoparticles were prepared via the ionic gelation method with slight modifications [52]. It was observed that as the amount of surfactant increased in the nanoparticle solution, the nanoparticles obtained were of smaller size with a lower polydispersity index. Regarding the effect of gelation temperature on the size of nanoparticles, a higher temperature, close to body conditions, seemed to provide better DLS results for the obtained nanoparticles compared with the ones prepared at room temperature.

The alginate nanoparticles were coated with a positively charged chitosan polymer to introduce functional groups on the surface for further conjugation studies and to positively charge the surface for better cellular uptake. Different molecular weights of the chitosan polymers, HMW (310–375 kDa, >75% deacetylated) and LMW (50–190 kDa, 75–85% deacetylated); different concentrations (1, 0.5, and 0.1% *w*/*v*); and different mixing ratios (1:1, 1:2, and 2:1 (CS:NP)) were investigated in this study to assess a suitable size and surface charge. Since an HMW chitosan has longer polymer chains, its viscous solution resulted in the agglomeration of nanoparticles, despite its positive surface charge (detected by zeta potential measurements). DLS results revealed that the LMW chitosan can cover the surface of particles without a significant size increase and with a more positive surface charge (Table 2). Curcumin molecules were loaded into the core alginate of the prepared nanoparticle to prevent a burst release and provide a more controlled drug release based on the swelling of the polymeric carrier. For both drug-loaded and empty nanoparticles, the chitosan coating resulted in an increase in surface charge and the obtained nanoparticles remained in the range of 230–260 nm, which is suitable to benefit from the EPR effect after their release into the circulation system all the way to their targeted tissue.

For this study, the nanoparticles were designed to be efficient carriers for antioxidants to ovarian tissue in particular, which is why the surface of the obtained nanoparticles was decorated with a folic acid molecule that can bind to folate receptors, which are over-expressed in ovarian cancer tissues in general. Free primary amine groups from the chitosan layer on the nanoparticles were successfully utilized for FA conjugation from its beta-carboxylic acid group through an amidation reaction. According to the DLS results, the FA conjugation did not substantially affect the size of nanoparticles; however, the decrease in the surface charge value indicates the conjugation of FA molecules onto the nanoparticles.

For the drug-loaded nanoparticles, the assembly was disturbed by dissociating the polymer–drug complex in the excess PBS/DMF mixture, and the amount of revealed drug molecules was measured by LC-MS/MS using the MRM method specifically prepared for curcumin molecular ion. As shown in Table 2, each step performed on the surface of nanoparticles resulted in a slight decrease in the amount of encapsulated drug at the alginate core (from 94% to 75%). However, the electrostatic interaction between the alginate surface and the chitosan layer may have ameliorated the stability of the nanoparticles and prevented burst drug release. By this strategy, the obtained core–shell nanoparticles bear a high potential to provide not only a sustained release of encapsulated drug molecules but also efficient delivery to the target.

**Figure 1:**
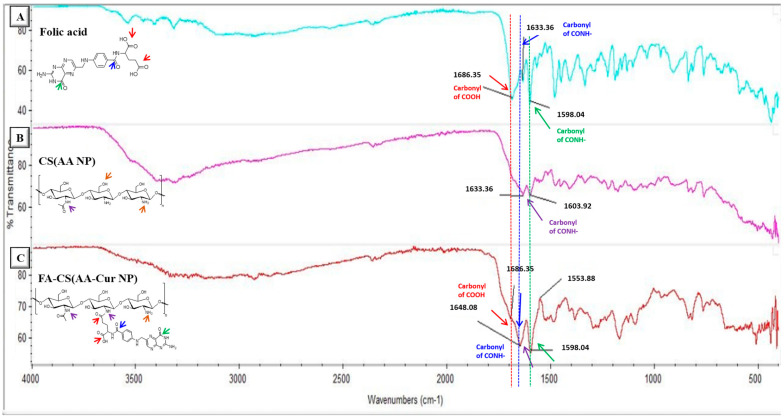
FT-IR spectra of FA molecule (**A**), CS(AA NP) (**B**), and FA-CS(AA-Cur NP)(**C**).

Chemical characterization of prepared curcumin-loaded and chitosan-coated nanoparticles was performed using FT-IR spectroscopy analysis. After purification of nanoparticles via the dialysis method, the solution was lyophilized to perform the analysis in powder form. It is clearly seen in Figure 1 that the intensity of the carbonyl peak (1633 cm^−1^) belonging to the primary amide increased for the FA-conjugated nanoparticle compared with the non-conjugated one. In addition, the newly formed peak at 1686 cm^−1^ for the FA-conjugated nanoparticles indicates the presence of a carboxylic acid group of FA incorporated onto the nanoparticle surface. Multiple peaks around 700–800 cm^−1^ (representing aromatic group bending in the spectrum of FA) are also detectable in the FA-conjugated nanoparticle spectrum. Moreover, characteristic peaks of alginate like –C–O–C– bonds (around 1050 cm^−1^) and -N-H peaks of chitosan polymer (around 3300 cm^−1^) can be observed in the spectrum of FA-conjugated nanoparticles.

**Figure 2:**
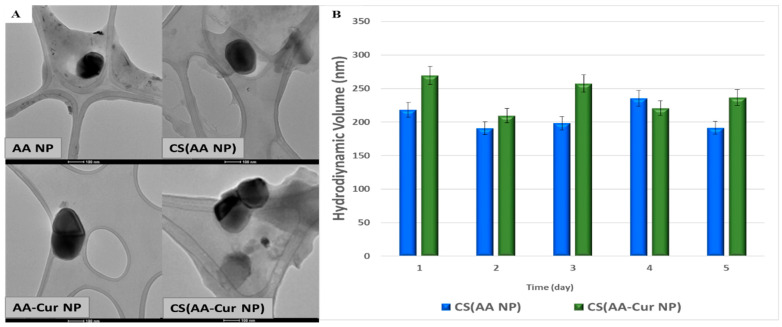
TEM images of prepared nanoparticles (**A**) and their stability behavior (**B**) (scale bar: 100 nm).

Morphological evaluation of the prepared nanoparticles was performed via TEM analysis. Figure 2A clearly illustrates the spherical shape of the nanoparticles. The empty nanoparticles appear to be a lighter color, whereas the drug-loaded particles can be distinguished with a dark color at the bottom. The stability behavior of the nanoparticles was evaluated by measuring their size change for 5 days. The comparative stability profiles of chitosan-coated empty and drug-loaded nanoparticles revealed a 15–20% change in size compared with that of their initial state (day 0) (Figure 2B), which might be attributed to their swelling and the dissociation of polymer chains from the nanoparticle structure.

**Figure 3:**
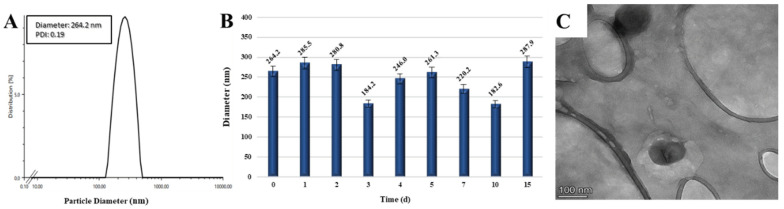
Size distribution (**A**), stability (**B**), and morphological evaluation (**C**) of FA-CS(AA-Cur NP).

Targeted nanoparticles with folic acid molecules conjugated to their surface were 264.2 nm in diameter with a symmetrical size distribution (Figure 3A). While the stability study revealed a slight increase in diameter due to their swelling in the aqueous media over 15 days of incubation (Figure 3B), the nanoparticles were observed to be spherical in shape in the TEM image (Figure 3C).

### 3.2. Characterization of Nanoparticle-Embedded Hydrogel Scaffolds (HG/CS(AA-Cur NPs))

#### 3.2.1. Morphological Characterization

**Figure 4:**
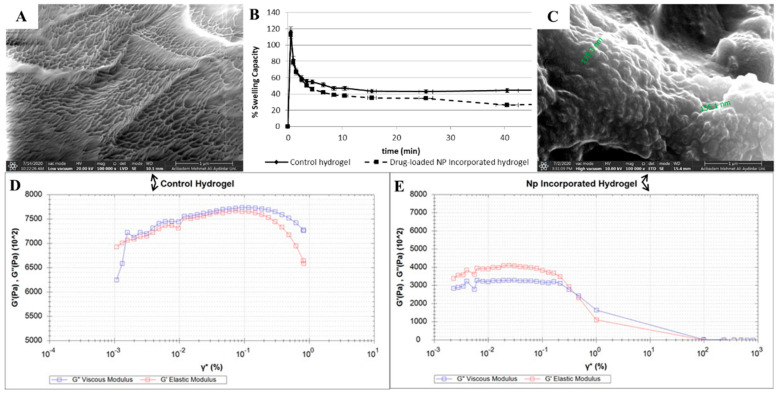
Morphological and mechanical evaluations for blank pNIPAM-based hydrogel (**A**,**D**), FA-CS(AA-Cur NP)-incorporated hydrogel (**C**,**E**), and their comparative swelling profiles (**B**).

The pNIPAM-based hydrogel scaffolds were prepared according to the literature procedures with slight modifications [34]. Gelation of thermo-responsive pNIPAM polymer chains was stabilized by cross-linking with a methacrylated ethylene-glycol-based monomer (TEG-MA), which was initiated based on the radical generation by APS and TEMED couple and fastened by increasing the temperature to human body temperature. Hydrogels without nanoparticles (control) were also prepared for comparison with the drug-loaded, nanoparticle-embedded hydrogel scaffolds. Morphological evaluation of the hydrogel scaffolds was performed by SEM analysis to confirm the porous structure. In Figure 4A, a lyophilized control hydrogel was observed to have a uniform porous structure, whereas the SEM image of a nanoparticle-embedded hydrogel scaffold confirms the presence of nanoparticles encapsulated during the gelation process (Figure 4C). A closer look at the SEM image shows the diameter of round-shaped nanoparticles inside the hydrogel to be in the range of 120–150 nm, which might be due to the shrinkage of nanoparticles due to the lyophilization process under high *vacuo*.

#### 3.2.2. Water Uptake Capacity

The swelling profiles of the hydrogels were investigated to determine their water uptake capacity over time and provide insight into their degradation behavior. Figure 4B demonstrates the percent swelling ratios of both the control and nanoparticle-embedded hydrogels scaffolds. A sudden and sharp increase in hydrogel weight was initially observed for both, followed by a sustained and slow decrease. This trend might be an indication of the slow degradation of obtained scaffolds, in spite of their less hydrophilic nature due to their pNIPAM chains and ethylene glycol units.

#### 3.2.3. Mechanical Characterization

The degradation behavior of control and nanoparticle-embedded hydrogel scaffolds were studied via an amplitude sweep test (Figure 4D and Figure 4E, respectively) using a rheometer. When a 0.001 to 100% strain was applied on the hydrogels, the nanoparticle-embedded hydrogel clearly demonstrated a linear viscoelastic behavior with a stiffness of 0.36 MPa up to 0.9% strain under 1 Hz frequency as a general method. Although the control hydrogel showed better stiffness at the beginning of the analysis, with a value of 0.70 MPa for elastic modulus, it switched to its yield point as the cross point at which G″ and G′ overlap at 0.005% strain, indicating a lack of structural integrity very quickly. The gelation profiles of these hydrogel scaffolds were also analyzed at three different frequencies (0.1, 50, and 100 Hz) that cover the stiffness range of human tissues [63]. These tests were applied to the hydrogel mixture solution, in which the strain was gradually increased. For the control hydrogel, as the frequency increased, stiffer gels were obtained, with increasing G′ storage modules value (Figure S1). Furthermore, for the nanoparticle-incorporated hydrogels, as the frequency of the analysis increased, loss and storage modules grew closer to each other compared with those of the control group, suggesting faster, better organization and assembly of the polymer chains to provide stiffer scaffolds, which is more obvious for the case at 100 Hz (Figure S2).

### 3.3. Drug Release Studies of Nanoparticles and Nanoparticle-Embedded Hydrogel Scaffolds

**Figure 5:**
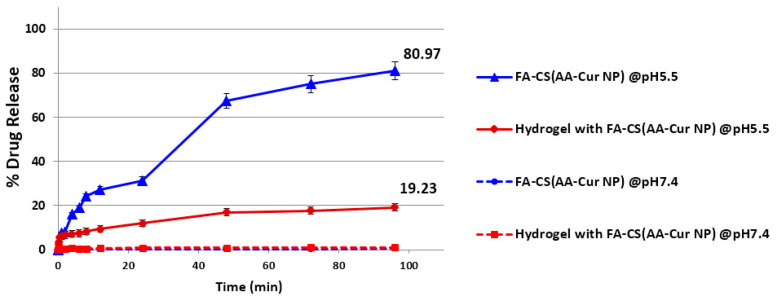
Release profiles of curcumin from NP alone and NP-incorporated hydrogels at different pH conditions.

The release profiles of loaded curcumin either from only nanoparticles or nanoparticle-embedded hydrogels were investigated at body temperature and under two different conditions with pH values of 7.4 and 5.5 to represent normal physiological conditions and inflammation at the tumor microenvironment, respectively. Based on the measured drug amounts that were released at different time points through the dialysis membrane, the percentages with respect to the initial loaded drug amounts were calculated. Figure 5 clearly reveals not only the effect of lower pH value but also the effect of hydrogel encapsulation on the drug release profile. It is well-known that an acidic environment leads to accelerated degradation of natural polymers, resulting in faster drug release. This is the case for both the nanoparticles themselves and their hydrogel-encapsulated versions. At a pH of 7.4, there was a negligible amount of drug released over 4 days, whereas at a pH of 5.5, the nanoparticles and nanoparticle-incorporated hydrogel appeared to liberate the drug molecules in a sustained manner, with almost four times more drug released from the free nanoparticles. This difference obviously originates from the fact that the encapsulation of the nanoparticles in the hydrogel scaffolds has a direct impact on the drug release profile such that the encapsulated drug molecules in the nanoparticles embedded in hydrogels were liberated through the hydrogel pores in a slower fashion, which may provide better control and a continuous supply of the drug payload.

### 3.4. Thermo-Responsiveness and Injectability of Nanoparticle-Embedded Hydrogel Scaffolds

**Figure 6:**
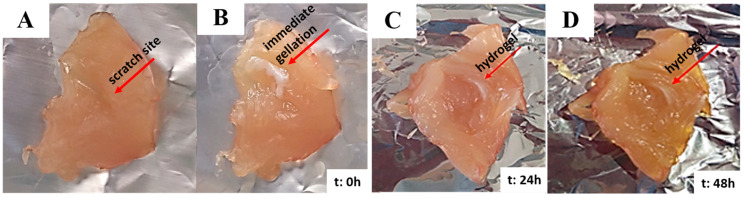
Scratch before the injection (**A**) and after the injection (**B**) of NP-incorporated, thermo-responsive pNIPAM hydrogel and its degradation behavior (**C**,**D**) on chicken breast tissue incubated at 37 °C.

Designed as an injectable hydrogel system, this scaffold was prepared via temperature-initiated gelation of pNIPAM chains and stabilized by simultaneous cross-linking with a methacrylate-based ethylene glycol monomer. Homogeneous mixing of this solution with drug-loaded nanoparticles provided encapsulation into the pores generated by polymer chains connected to each other by a cross-linked network of ethylene-glycol-based monomers to form the resultant hydrogel scaffold. Together with the abovementioned characterization of hydrogels prepared with or without nanoparticles, their thermo-responsive feature was evaluated via an ex vivo-like condition generated by freshly obtained chicken breast tissue (4 cm × 4 cm). At body temperature, a deep scratch was made on the tissue sample, followed by the application of nanoparticle-containing hydrogel solution with a syringe. As expected, gelation at 37 °C occurred within 30 s (Figure 6), and the generated hydrogel covered the scar completely. Moreover, incubation of this assembly was continued for 2 days at body temperature to assess the stability of the obtained scaffold at the scratch site, positively contributing to its ameliorating effect on wound closure.

## 4. Conclusions

The nanoparticle-incorporated hydrogels prepared in this study were designed as effective drug delivery systems with multifunctional features, including rapid injectability at body temperature, slower drug release profile, and direct targeting to cancerous cells with over-expressed folate receptors. For this purpose, curcumin was selected not only for its anti–carcinogenic but also for its anti-inflammatory activity. Alginate nanoparticles with an encapsulation efficiency of ~94% were loaded with curcumin to create a drug reservoir core, which was coated with another natural polymer, chitosan, to increase its stability. By benefiting from the functional groups of chitosan, folic acid was conjugated to the surface of these core–shell nanoparticles, providing targeted and drug-loaded carriers with a diameter (264.2 nm) suitable for the EPR effect. Incorporation of these nanostructures into the pNIPAM-based hydrogels with a swelling ratio of approximately 70% provided fast injectability (within 30 s) against a freshly obtained chicken breast tissue and demonstrated an almost four times slower drug release profile compared with that of free nanoparticles in slightly acidic media, which mimics the tumor microenvironment. The incorporation of nanoparticles clearly contributed to prolonged viscoelastic behavior. In conclusion, these nanoparticle-integrated hydrogel scaffolds have the potential to serve as efficacious therapeutic tools for delivering drug molecules to tumor sites with a simple injection, post-operational procedures, and wound closure.

## Figures and Tables

**Table 1:** Preparation conditions for nanoparticles.

No *	CaCl_2_ (50 mM) (mL)	NaAlg (0.5%) (mL)	L-Lysine (0.1%) (mL)	SDS (0.1%) (mL)	Temp (°C)
1	4.75	0.25	2	2	25
2	4.75	0.25	2	2	40
3	4.75	0.25	3	3	25
4	4.75	0.25	3	3	40

* All conditions were applied as using 0.5% (*w/v*) NaAlg and 50 mM CaCl_2_.

**Table 2:** Characterization details of prepared nanoparticles.

Nanoparticle	Hydrodynamic Volume (nm) ^1^	PDI ^1^	Zeta Potential (mV) ^2^	EE% ^3^
AA NP	249.2	0.79	−0.4 ± 0.4	-
CS (AA NP)	261.4	0.64	1.7 ± 0.6	-
AA-Cur NP	233.1	0.67	−2.1 ± 0.4	94
CS (AA-Cur NP)	245.2	0.75	1.9 ± 0.5	88
FA-CS(AA-Cur NP)	264.2	0.19	0.8 ± 0.3	75

^1^ Size and PDI values were measured in ddH_2_O by DLS instrument. ^2^ Surface charge values were measured using 1:10 dilution factor (*v/v*) in 1 mM NaCl. ^3^ Encapsulation efficiency values were measured by LC-MS/MS instrument.

## Data Availability

Not applicable.

## Supplementary Materials

The following supporting information can be downloaded at https://www.mdpi.com/article/10.3390/pharmaceutics15092358/s1, Figure S1: Mechanical evaluations for FA-CS(AA-Cur NP)-incorporated hydrogel under different oscillation pressures, 0.1 Hz (A), 50.5 Hz (B), and 100 Hz (C); Figure S2: Degradation of drug-loaded NP-embedded hydrogel and its comparison with empty NP-embedded hydrogel, 24 h after their injection into tissue sample.
