# Supplementary material for: Thermo-Responsive Hydrogels Encapsulating Targeted Core–Shell Nanoparticles as Injectable Drug Delivery Systems
Source: Pharmaceutics. 2023 Sep 21;15(9):2358. doi: 10.3390/pharmaceutics15092358 (PMC10537279; doi:10.3390/pharmaceutics15092358)
Supplement: Supplementary file 1 [file pharmaceutics-15-02358-s001.zip › pharmaceutics-2579641-supplementary.pdf]

pharmaceutics

Supporting Information

Thermo-Responsive Hydrogels Encapsulating Targeted Core–Shell Nanoparticles as Injectable Drug Delivery Systems

Elif Gulin Ertugral-Samgar [1], Ali Murad Ozmen [1] and Ozgul Gok [1,2,*]

[1] Medical Engineering Program, Graduate School of Natural and Applied Sciences, Acibadem Mehmet Ali Aydinlar University, 34752 Istanbul, Turkey; elif.ertugral@live.acibadem.edu.tr (E.G.E.-S.); alimurad93@gmail.com (A.M.O.)
[2] Department of Biomedical Engineering, Faculty of Engineering and Natural Sciences, Acibadem Mehmet Ali Aydinlar University, 34752 Istanbul, Turkey
* Correspondence: ozgul.gok@acibadem.edu.tr; Tel.: +90-216-5004188

Appendices& Supplementary Materials

Appendix S1. Mechanical Evaluation of NP-Incorporated Hydrogel Scaffolds…………………………………..………2

Figure S1. Mechanical evaluations for FA-CS(AA-Cur NP) incorporated hydrogel under different oscillation pressure, 0.1 Hz (A), 50.5 Hz (B) and 100 Hz (C) ………………………………………………………………………………………2

Figure S2. Degradation of drug-loaded NP embedded hydrogel and its comparison with empty NP embedded hydrogel, 24h after their injection into tissue sample………………………………………………………………………..………2

Appendix S1. *Mechanical Evaluation of NP-Incorporated Hydrogel Scaffolds*

Gelation tendency of prepared hydrogels was investigated with a rheometer. In Figure S1, G'' and G' values represent elastic modulus and viscous modulus, respectively. Since the injectability rate of prepared scaffolds also depends on the mechanical properties of the tissue into which it is placed, how the nanoparticle-polymer mixture solution behave under different pressure values becomes very significant to investigate. With this motivation, the resultant nanoparticle-incorporated hydrogel solution was evaluated with a reometer for its mechanical properties at different frequency values as 0.1, 50.5 and 100 Hz. While NP incorporated solution turns into a gel form immediately under 0.1 Hz, it seems to keep its integrity up to almost 5 minutes. On the other hand, as the applied frequency was increased to 50.5 Hz (the average value of the applied frequency range), the viscous modules started to appear closer to the elastic one at the beginning of the experiment which indicates the immediate gelation and the difference between them gets smaller for a longer period of time, which points to the increased stability of the obtained hydrogel under a certain amount of pressure generated by a frequency of 50.5 Hz, which is the one similar to the human tissues.[1] Moreover, it was seen in Figure S2C thatthe nanoparticle incorporated polymer solution turned into a complete gel form under 100 Hz even at the beginning of the experiment withan obvious difference between G' and G'' values upon temperature increase to 37 °C. Both modulus values stayed stable during the whole measurement period, which points to the prolonged integrity of the obtained hydrogel scaffold compared to the control one without nanoparticles inside.

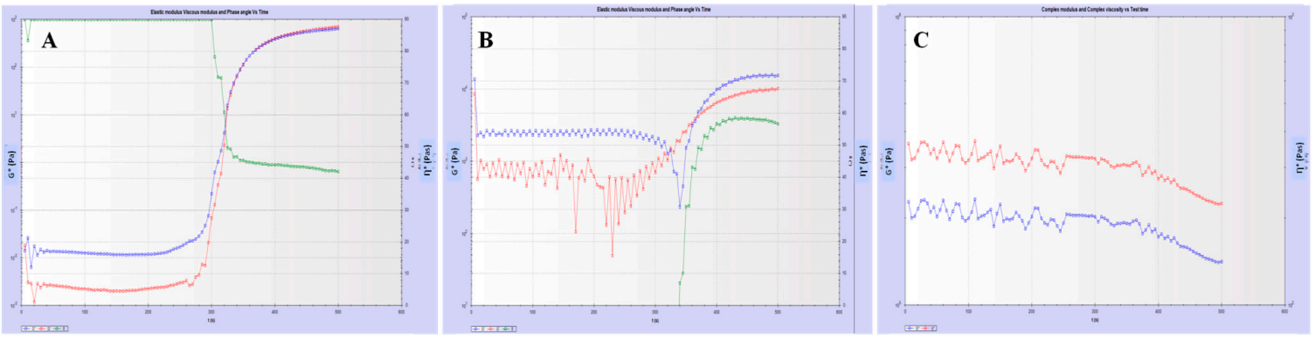

Figure S1. Mechanical evaluations for FA-CS(AA NP) incorporated hydrogel (as control) under different oscilation pressure, 0.1 Hz (A), 50.5 Hz (B) and 100 Hz (C).

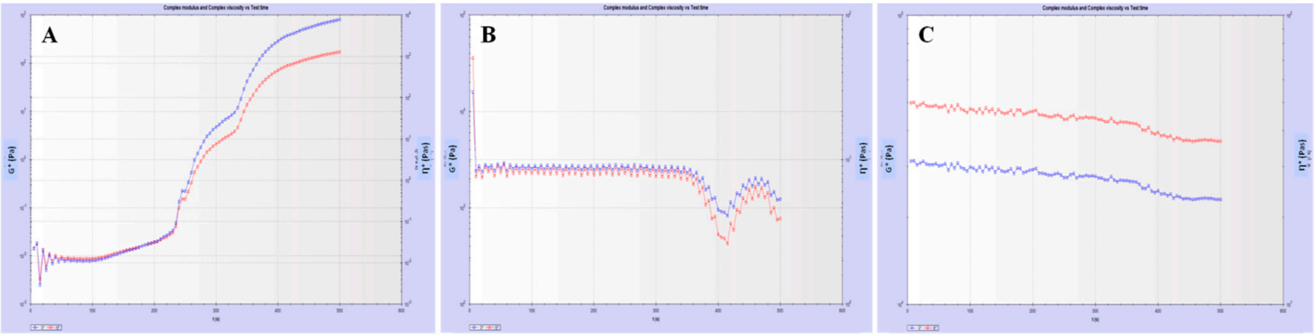

Figure S2. Mechanical evaluations for FA-CS(AA-Cur NP) incorporated hydrogel (drug containing) under different oscilation pressure, 0.1 Hz (A), 50.5 Hz (B) and 100 Hz (C).

References

(1) Matsuzaki, S. Mechanobiology of the Female Reproductive System. *Reprod Med Biol.* **2021**, 371–401.
